# Voices of Polymedicated Older Patients: A Focus Group Approach

**DOI:** 10.3390/ijerph17186443

**Published:** 2020-09-04

**Authors:** Ana Isabel Plácido, Maria Teresa Herdeiro, João Lindo Simões, Odete Amaral, Adolfo Figueiras, Fátima Roque

**Affiliations:** 1 Research Unit for Inland Development, Polytechnic of Guarda (UDI-IPG), 6300-559 Guarda, Portugal; anaplacido@ipg.pt; 2 Institute of Biomedicine (iBiMED) and Department of Medical Sciences, University of Aveiro, 3810–193 Aveiro, Portugal; teresaherdeiro@ua.pt; 3 Institute of Biomedicine (iBiMED) and School of Health Sciences, University of Aveiro, 3810–193 Aveiro, Portugal; jflindo@ua.pt; 4 Health Sciences School, Polytechnic of Viseu, 3500–843 Viseu, Portugal; mopamaral@gmail.com; 5 Department of Preventive Medicine and Public Health, Faculty of Medicine, University of Santiago de Compostela, 15702 Santiago de Compostela, Spain; adolfo.figueiras@usc.es; 6 Consortium for Biomedical Research in Epidemiology and Public Health (CIBER en Epidemiología y Salud Pública-CIBERESP), 28001 Madrid, Spain; 7 Institute of Health Research of Santiago de Compostela (IDIS), 15706 Santiago de Compostela, Spain; 8 Health Sciences Research Centre, University of Beira Interior (CICS-UBI), 6200–506 Covilhã, Portugal

**Keywords:** polypharmacy, home-dwelling older patients, medication errors, focus group sessions

## Abstract

Polypharmacy in older adults is frequently associated with incorrect management of medicines, which causes drug-related problems and, subsequently, poor health outcomes. Understanding why older adults incorrectly manage their medicines is fundamental to health outcomes, however, it is an issue that remains poorly explored. The aim of this study is to examine older people’s perceptions, attitudes, beliefs, and concerns in the central region of Portugal. Thirteen focus groups with sixty-one older adults taking five or more prescription medicines were conducted to explore older patients’ perceptions and beliefs about and management of their medication. Sampling was conducted until theme saturation had been achieved. Transcripts were coded and data were obtained using the NVivo qualitative data-analysis software programme. Older adults recognise the importance of medicines for ensuring healthy ageing. Owing to a lack of literacy, however, they frequently commit medication mistakes and compromise their health outcomes. Promoting the literacy and empowerment of older patients, as well as strengthening the relationship between health professionals and patients, is crucial when it comes to addressing drug-related problems and improving health outcomes.

## 1. Introduction

In the 21st century, the evolution of health technologies has contributed to the improvement in health outcomes and overall healthcare system performance [1]. Today, the world population lives longer, albeit not necessarily better, with greater autonomy and independence [2]. Getting older is frequently associated with multiple comorbidities, polypharmacy, and cognitive impairment, which may increase the likelihood of drug-related problems (DRPs) [3].

When correctly handled, medicines are fundamental technologies for achieving better health outcomes [4,5]. The increased fragility of the older population can lead them to suffer the consequences of medication errors in the form of adverse drug effects or therapy failure, and ultimately increased morbidity and mortality [3,6]. In older people, factors such as polypharmacy, socio-economic conditions, and perceptions of disease and treatment also seem to be related to DRPs [7]. Hence, older adults’ attitudes, opinions, and concerns and how they manage their medicines can be crucial when it comes to designing strategies aimed at increasing our knowledge of routine medicine management and improving the quality of care and health outcomes.

Qualitative focus group studies could be a key research tool for this purpose. In contrast to other methods (interviews, questionnaires), focus groups can identify all the dimensions of a problem, even those that are unexpected, while also being extremely suitable for age strata with low literacy [8]. Even so, we were unable to locate any focus group study on this subject. Accordingly, the designated aim of this study was to explore home-dwelling older patients’ perceptions, attitudes, beliefs, and concerns about their medicines.

## 2. Materials and Methods

### 2.1. Ethical Approval

This study obtained the ethical approval of the Central Regional Health Administration (*Administração Regional de Saúde do Centro IP/ARS-C*) (registry no. 105/2017) and the authorisation of the respective health centre directors.

### 2.2. Design

A focus group (FG) approach was conducted to stimulate polymedicated patients who might otherwise not be used to discussing the subject, in order to share their opinions and concerns about daily management of medications. The FG sessions were moderated by a researcher (F.R.), following a topic guide (see Supplementary data, Supplement 1) based on a review of the literature and drawn up by a group of pharmacists and epidemiological experts [3].

### 2.3. Setting

The FG sessions took place at 13 public health centres falling under the Central Regional Health Administration and encompassing a total of 40,835 registered older patients (age ≥ 65 years). Public health centres are the gatekeeper of Portugal’s National Health Service (NHS). The NHS is predominantly financed by taxes and some out-of-pocket payments that include co-payment for a wide range of services, though there are income-based exemptions for certain population groups (and certain medical conditions) [9,10]. Older adults with an average salary 1.5 times below the value of the social support index are exempt from co-payment for any publicly provided services [11]. However, the bulk of co-payments are made for pharmaceutical products, to which different levels (ranging from 15% to 90%) of co-insurance are applied according to their therapeutic value [9].

### 2.4. Holding of Focus Group Sessions

Potential participants were home-dwelling patients aged 65 years or over who were taking five or more medicines. People with mental conditions that precluded them from reacting appropriately and those who lived in nursing homes were excluded from the study.

Participation was voluntary, without inducements of any kind, and all patients signed an informed consent form before taking part in the meeting. Information on each participant’s prescribed medication was provided by electronic records or obtained via the patient’s treatment guide.

Thirteen FG sessions were held from May to October 2018, and lasted for 60–90 min. They were carried out until saturation of information was reached on the research questions. Before the beginning of each session, the moderator, an experienced researcher in FG studies, reminded the participants of the study goals and the fact that audio recordings were being made of the sessions. The moderator ensured the participants that the content matter would remain confidential at all times and that the data would be processed without identification of participants.

### 2.5. Analysis

All the sessions were transcribed and coded with a serial number by one researcher (A.I.P.). To ensure trustworthiness, one month after the initial hearing, the same researcher replayed the tape and reviewed the content of the transcription.

To obtain a thematic description of the data, an inductive thematic analysis with open codes was performed, using the NVivo qualitative data-analysis software programme (QSR International’s NVivo version 12 qualitative data analysis software, from SR International Pty Ltd. nVivo. Doncaster: QSR International Pty Ltd; 2019, Doncaster, England). In a first stage, A.I.P. and F.R. read through the FG transcripts a number of times to facilitate familiarisation with the data. Initial codes were then generated and clustered to enable a search to be made for themes. At this point, themes were reviewed, and relevant data and interpretation of findings were discussed by the research team. Lastly, the themes were named and a report was made in accordance with the checklist of consolidated criteria for reporting qualitative research (COREQ; see Supplementary Data, Table S1) [12]. The list of prescription medicines was converted to the corresponding Anatomical Therapeutic Classification (ATC) code, using the World Health Organisation (WHO) Collaborating Centre for Drug Statistics Methodology website [13].

## 3. Results

A total of 61 participants, distributed across 13 FGs, were enrolled in this study. The mean (±SE) age of the participants was 76.3 ± 0.77 years, and 47.5% were women (Table 1). The mean number (± SE) of medicines per patient was 8.5 (min 5, max 16). FG participants’ medication profiles are shown in Table 2. The following three main themes emerged from the FG data: (i) daily medicine routine; (ii) beliefs and attitudes regarding medicines; and (iii) relationship with health professionals (Table 3).

FG participants’ medication profiles are shown in Table 2.

The following three main themes emerged from the FG data: (i) daily medicine routine; (ii) beliefs and attitudes regarding medicines; and (iii) relationship with health professionals (Table 3).

### 3.1. Daily Medicine Routine

Patients identified medicines as part of their daily routine. Within this theme, three subthemes emerged, that is, “knowledge of medicines”, “handling medicines and administration schedules”, and “barriers to medication adherence”.

#### 3.1.1. Knowledge of Medicines

FG participants expressed no doubts about recognising their medicines because they “have been taking them for a very long time” (FG10P1). Some of them saw the ability to read as being reason enough to identify their medicines correctly: “I can read” (FG1P2)… “me too” (FG1P1). Only a few participants admitted that they experienced difficulties in recognising their medicines: “I don’t recognise them very well” (FG3P6). However, the majority of them did not refer to their medicines by name, but instead mentioned the medical condition associated with the pills, for example, “I take 2 for diabetes, 1 for cholesterol, 1 for the head…” (FG1P1), or the colour/shape of the pills, for example, “a yellow pill” …. “all morning pills have the same shape… (FG4P1), and only a small number associated the medicine’s name with the pathology or the colour of the medication.

#### 3.1.2. Handling Medicines and Administration Schedules

Most of the participants said that they could handle their medicines without help: “I don’t have anyone” (FG12P2) … “me neither” (FG12P3). Only a few participants admitted that they were incapable of managing their medicines correctly, and that they had the family’s help: “We, have our daughter’s help” (FG12P4); “If I didn’t have the help of my wife, I’d have problems” (FG11P3). Medicines were stored according to their daily routine: “morning pills I put in the kitchen and night pills I put in the bedroom” (FG7P3). The majority mentioned that they had a plastic bag in which they put all the pillboxes, as well as a box for the specific day/week: “… I have a box for the week…” (FG 9P5); “… oh! I don’t want a box for the week, I have a box for the day” (FG 9P1). Patients who used the medicine box admitted that they removed the pill from the blister and did not recognise the medicines inside the box. It was for this reason that, if they forgot to take a pill, they were then unable to identify which one they had forgotten to take: “If I don’t take it, it stays here in the box… look, this week I’ve missed two so far …” (FG10P2).

#### 3.1.3. Barriers to Medication Adherence

Participants acknowledged the importance of medication and said that they were complying with their treatment regimens. During the discussion, however, some of them said, “instead of taking it every day, I take it every other day…” (FG11P5). Barriers to medication adherence appear to be related to personal issues associated with daily life, for example, patients admitted that, if a tablet interfered with their daily routine, they rescheduled their medication, or did not take it: “… If we go out for lunch… sometimes, it’s not that I forget, but it’s because I just don’t take it” (FG11P2). Other participants admitted that they knowingly missed medicines, with uncomfortable effects: “… I couldn’t go out; I was always running to the bathroom to pee… sometimes I wet myself” (FG1P2). Medication adherence is also affected by the price of medicines: “…I’ve already seen patients who don’t buy their prescribed medicines because they’re too expensive” (FG4P2). Some participants admitted that there were times when they did not take medicines for fear of a hypothetical adverse drug reaction described in the drug prospectus: “I read the package leaflet, the medicine is harmful to so many things… I only took the pills for two days” (FG3P6). They also believe that they can adjust the treatment without their primary care physician’s consent, and that this behaviour does not affect their health outcomes: “Sometimes I don’t take the pills…Today I missed the pills… Sometimes I can take a break, there’s no problem” (FG8P1). Some patients admitted that, sometimes, they missed taking the pills because they forgot and felt that this was not an issue: “… sometimes I forget, but there’s no big problem…” (FG9P1). They maintain that they are more compliant with certain medicines, such as painkillers, because they feel the lack of the medication’s effect: “Sometimes I forget but I feel the pain, and then I remember and I take it” (FG8P2). Otherwise, pills associated with painless conditions (e.g., cholesterol) are easily forgotten: “... I sometimes forget to take the cholesterol medicine” (FG8P1).

When it came to strategies used to decrease DRPs associated with medication errors, participants said that they did not need a strategy. Yet almost all admitted to using a pillbox to avoid forgetting or having some method or scheme to remind them to take their medicine. Participants also insisted that they had no difficulty in taking medicines or in dealing with multiple medicines. However, they often remarked that they sometimes reduced the dosage of a particular drug because they thought that they were taking too many pills: “I think I’m taking too many pills and that my body will explode someday …. / For instance, I’m taking a prostate pill, I can’t take 2 every day because I think that two pills are too many … / because I also take pills for diabetes and heart…” (FG8P3).

### 3.2. Beliefs and Attitudes about Medicines

Participants seem to be overwhelmingly resigned to the need to take their medicines, and described medicines as part of their routine, “… I mean, it’s already become a habit” (FG3P1), and daily life, “… in the morning, after waking up, I get breakfast ready and take 3 pills” (FG1P1). They also stated that a daily routine was essential to remembering to take their medicine: “… I know which pill is for hypertension, … I take it in the morning on an empty stomach …. This morning I didn´t take it… I forgot… When I got here, oh! I skipped my hypertension pill” (FG1P2); “… I´m going to cook my husband’s meal… oh! I skipped the pill” (FG1P2). Older patients’ behaviour is influenced by their beliefs and by the importance that they attach to each medicine. They have the perception that medicines are essential for ensuring their well-being and preventing disease, and feel that all medicines are equally important for ensuring their well-being. However, patients were perceived as managing their medicines according to the severity of the disease, that is, they frequently talk about their heart medicines and their cardiologist appointments: “the cardiologist said it’s a problem in the aorta” (FG11P1),” “…yes, the cardiologist said so” (FG12P3). It was also observed that older patients were influenced by television commercials and by their family and friends: “the doctor prescribed me calcium and I took it for a long time. But then I saw the ad on the telly, my sister took it and felt good, so I started taking it too, and I’m feeling very well” (FG9P1). Attitudes to medication compliance seem to be influenced by their knowledge of herbal products: “…my daughter makes tea with 3 olive leaves, and isn’t taking any medicine for arterial pressure” (FG2P4). Participants had difficulties in admitting that they used herbal products. They described the effect of herbal teas, but concluded by saying, “I never drink it” (FG3P1) or “it’s a friend or a relative that drinks it, not me” (FG2P1), or “My brother took it every day” (FG9P3). There is one exception when participants talk about prostate disease; they often say, with some pride, that they replaced their prostate medicine with a herbal product and felt better, “I never took a pill… I drink tea…” (FG2P5). 

Some participants believe that generic medicines are equal to reference medicines, and have the advantage of lower prices, “Generic medicines are cheaper than branded ones” (FG7P6); other participants did not trust generic medicines and preferred to spend more money than take a generic, “I believe that generic medicines are not as good as branded medicines” (FG4P2), “Branded medicines are more expensive but we do everything for health” (FG6P1). Participants frequently referred to generic medicines as medicines in a different box with a different name, which could cause some mistakes, such as duplication: “I didn’t feel anything but the pharmacists told me that if I was taking that pill I couldn’t take this one … The names are different, but they do the same thing” (FG10P1).

Patients did not have the perception that medicines should be taken with water. In many cases, they took their medicines with food or without any pill-swallowing aids, and a few admitted to occasionally taking their medicine with alcohol. “…in the morning I take my medicines with coffee” (FG7P2), “… I’m having soup, so I take my pills with soup” (FG9P2), “… at lunch I take my pills with wine” (FG13P1).

### 3.3. Relationship with Health Professionals

Patients have the perception that health professionals play a supportive role and foster a sense of trust. Patients showed their trust in health professionals and believed that health professionals are essential to ensuring correct management of medicines and decreasing DRPs: “We do exactly as the doctor says” (FG1P2); I can’t take a pill without going to the doctor” (FG2P1). “We talk to the pharmacist… and he writes down the instructions for the pills on the box” (FG4P1). During the course of the discussion, however, patients frequently stated that they did not inform the doctor about the interruption of medicines, alterations to their prescription, or the introduction of supplements or herbal products: “I never tell the doctor that I’m taking this medicine” (FG9P1); “My physician doesn’t agree but I take it all the same...” (FG11P2); “I interrupt one medicine…/I don’t tell the doctor” … I put it in the garbage” (FG 1P1); “Once I tried to reduce the dosage of my diabetes pills” (FG6P3). 

## 4. Discussion

To our knowledge, this is the first focus group study to explore older polymedicated adults’ perceptions, beliefs, and attitudes about the management of their medicines. Older patients’ desire to maintain a good quality of life and have the perception that the proper use of medicines is essential to achieving this and enjoying “healthy ageing”. To this end, they frequently develop strategies to decrease medication errors and prevent possible DRPs. Nevertheless, their efforts may not be altogether successful because they do not acknowledge the need to comply with the treatment regimen to ensure good health outcomes.

Although the Portuguese healthcare system is ranked as the 12th best in the world [14], only 9% of older Portuguese patients are considered healthy, a worrisome figure when compared with Switzerland, Germany, Austria, or France, where the percentage of healthy older adults is 51%, 38%, 58%, and 37%, respectively [15]. Considering that the majority of the older Portuguese adults enrolled in this study were unaware of the value of complying with their treatment regimens to ensure good health outcomes, our results attest to the fact that Portuguese older adults’ lack of knowledge could be a major contributor to their unhealthy status and show that health literacy is a cornerstone of healthy ageing.

To reduce medication errors, FG participants develop external and/or internal memory strategies [16]. External memory strategies tend to be effective for new tasks and unfamiliar objects and less effective for repetitive tasks when the object in question becomes more familiar [16]. Internal memory strategies, such as associating medication with a specific task, are highly dependent on the daily routine, and so any change in that routine is reason enough to give rise to a medication error [17]. Medicine boxes were very popular among the FG participants. It is thus our considered opinion that, before recommending the use of such boxes, health professionals should ensure that patients understand the correct way in which they should be handled; this opinion is in line with a previous study [18].

Older adult FG participants had the perception that there no doubts about recognising their medicines because they had been taking them for a long time. However, previous studies [19,20] concluded that there is no relationship between the number of years in treatment and a subject’s ability to remember the name, treatment, or precautions to be taken when using medicines. Our study showed that, rather than referring to the name or the precautions to be taken when handling their prescribed medication, participants frequently referred to the colour of the pills or the reason for taking them, suggesting a lack of knowledge of their prescription medicines; these observations are in line with those of a previous study [20].

Owing to the lack of standardisation of pill form/colour and drug packaging, a change of pharmaceutical laboratory is reason enough for older adults to commit treatment duplication errors. This could pose a problem in a case where a reference medicine is replaced by a generic. We observed that some patients mistrust generic medicines, an observation corroborated by Bulsara et al. [21]. We thus feel that these two weaknesses might well offset the advantage of better adherence owing to the latter’s lower price. It would thus be advisable for pill form and colour to be standardised.

Our study showed that patients frequently argue that they take too many medicines, and thus try out medicines and weigh the risks and benefits. This erratic behaviour pattern cuts across all ages and is referred to by some as a “drug holiday” [22]. Patients believe that flexibility in adhering to their medication regimen allows life to continue without too many disruptions [22]. Older adults with fewer resources were also observed to be less compliant and to skip doses in order to make their medicines last longer. This type of behaviour has also been previously described [23,24].

FG participants showed that fear of an adverse event is a reason for interrupting treatment. This fear seems to be related to a lack of comprehension of the drug prospectus; a finding that is in line with the literature [25].

Some older adults reported that they regularly consumed *herbal products* and, in some cases, had quit their prescription medicines and were taking herbal teas, something that can boost drug–drug interactions [26]. In our study, the most frequently mentioned herbal products were ginkgo biloba, olive tree infusion, and infusions to decrease prostate problems.

Our study observed that older patients are influenced by pharmaceutical commercials and take these products without discussing the need to use them with a health professional. Such impulsive behaviour may well imply that pharmaceutical commercials have a negative influence on the doctor–patient relationship, thereby promoting a progressive lack of trust by patients in their primary care physician, and ultimately compromising WHO recommendations [27]. Overall, FG patients trust in their health professionals, but admit to concealing some behaviour, which can influence treatment efficacy. Bearing in mind that older adults place enormous value on trust, a good relationship between health professionals and older adults can act as the linchpin for enhancing adherence to treatment and satisfaction with the healthcare system [28,29].

This study has some limitations linked to the chosen methodology. FG participants were selected by their primary care physician because these patients’ behaviour could be better than average in terms of drug compliance. To reduce this type of bias, each FG session had at least some patients selected by different primary care physicians. Despite the fact that patients may follow the general trend to give similar answers, group discussions may prove difficult to steer and control, and relevant topics may be missed; this methodology’s generalisability is best judged in terms of logical inference and credibility of analysis [30].

The results of this FG study are useful for designing didactic material to address the main DRPs observed in older adults, demystify false beliefs, and promote correct management of medicines.

## 5. Conclusions

Older adults attach great value to their medicines, yet nevertheless have positive and negative perceptions of and attitudes to polypharmacy. These attitudes reflect the ambivalence of feeling grateful for the existence of medicines that alleviate symptoms and improve their life expectancy, while being afraid of adverse reactions and having doubts about the need to use some medicines. Promoting the health literacy and empowerment of older patients and strengthening the doctor–patient relationship are both essential for dispelling false beliefs and enhancing health outcomes among polymedicated older patients.

## Figures and Tables

**Table 1:** Socio-demographic characteristics of participants.

Participants (N = 61)	N (%)
Female	29 (47.5)
Age (mean ± SE)	76.3 ± 0.77
65–69	9 (14.8)
70–74	17 (27.9)
75–79	18 (29.5)
80–84	13 (21.3)
85–89	3 (4.9)
≥90	1 (1.6)

**Table 2:** Use of medicines in Anatomical Therapeutic Classification (ATC) pharmacological groups A, C, and N.

ATC Pharmacological Groups				N (%)	Mean Per Patient
**A- ALIMENTARY TRACT AND METABOLISM**				119 (20)	2.38
**A02B Drugs for peptic ulcer and gastro-oesophageal reflux disease (GORD)**				40	
			A02BA H_2_-receptor antagonists	2	
			A02BC Proton pump inhibitors	35	
**A10B Blood-glucose-lowering drugs, excl. insulins**				21	
			A10BA Biguanides	8	
			A10BD Combinations of oral blood-glucose- lowering drugs	5	
**C- CARDIOVASCULAR SYSTEM**				85 (15.0)	1.7
**C03C High-ceiling diuretics**				27	
			C03CA Sulphonamides, plain	16	
**C09D Angiotensin II receptor blockers (ARBs), combinations**				21	
			C09DA Angiotensin II receptor blockers (ARBs) and diuretics	6	
			C09DB Angiotensin II receptor blockers (ARBs) and calcium channel blockers	6	
**C10A Lipid modifying agents, plain**				34 (16.7)	
		C10AA HMG CoA reductase inhibitors		21	
**N- NERVOUS SYSTEM**				59 (10.3)	1.18
**N02B Other analgesics and antipyretics**				22	
			N02BA Salicylic acid and derivatives	7	
			N02BE Anilides	8	
**N05B–Anxiolytics**				23	
			N05BA Benzodiazepine derivatives	19	

**Table 3:** Major themes from focus groups.

Category of Themes	Category of Subthemes	Coding Concepts
**Daily medicine routines**	Knowledge of medicines	Identification of medicines Association between medicines and pathology Identification of difficulties
	Handling medicines and administration schedules	Handling ability Storage Strategies
	Barriers to medication adherence	Compliance Medication errors Lack of knowledge Self-medication Strategies to reduce drug-related problems
**Beliefs and attitudes regarding medicines**		Importance of medicines Living with medicines Fears Influences Generic medicines
**Relationship with health professionals**		Importance of health professionals Communication Trust

## Supplementary Materials

The following are available online at https://www.mdpi.com/1660-4601/17/18/6443/s1, Supplementary data S1: FOCUS GROUP GUIDE; Table S1: Checklist Coreq.
